# BUILDING CAPACITY: GRECCS’ CONTRIBUTION TO SPECIALIZED GERIATRIC WORKFORCE TRAINING

**DOI:** 10.1093/geroni/igae098.0901

**Published:** 2024-12-31

**Authors:** Marianne Shaughnessy

**Affiliations:** Department of Veterans Affairs, Middle River, Maryland, United States

## Abstract

In 1975 Congress established Geriatric Research, Education, and Clinical Centers (GRECCs) within the Veterans Health Administration as Geriatric Centers of Excellence. Twenty GRECCs currently exist across the United States that bring together diverse interdisciplinary teams of researchers, educators and clinicians. All GRECCS are also associated with major academic research universities. Due to their unique nature, GRECCs are well positioned to advance the training and development of the scientific and clinical geriatric workforce across health care disciplines and settings. Their environments foster learning for trainees engaged in health professions careers from initial training through advanced fellowships. As Geriatric Centers of Excellence, GRECCs also provide educational programs and resources to train VA healthcare providers in geriatric principles and best practices. This presentation will include an overview of the GRECC programs, their areas of specialty, and clinical and didactic training opportunities offered. A discussion of GRECC collaborations with academic institutions and community partners to disseminate geriatric education and promote geriatrics will also be discussed. Education, Research, and Clinical training delivered through the GRECCs help to ensure that clinicians and researchers have the knowledge and skills necessary to develop and deliver high-quality, patient-centered care to older adults.

